# Phenotype and Distribution of Immature Neurons in the Human Cerebral Cortex Layer II

**DOI:** 10.3389/fnana.2022.851432

**Published:** 2022-04-08

**Authors:** Simona Coviello, Yaiza Gramuntell, Patrycja Klimczak, Emilio Varea, José Miguel Blasco-Ibañez, Carlos Crespo, Antonio Gutierrez, Juan Nacher

**Affiliations:** ^1^Neurobiology Unit, Program in Neurosciences and Institute of Biotechnology and Biomedicine (BIOTECMED), Universitat de València, Burjassot, Spain; ^2^Spanish National Network for Research in Mental Health, Centro de Investigación Biomédica en Red de Salud Mental (CIBERSAM), Madrid, Spain; ^3^Unidad de Cirugía de la Epilepsia, Hospital Universitario La Fe, Valencia, Spain; ^4^Fundación Investigación Hospital Clínico de Valencia (INCLIVA), Valencia, Spain

**Keywords:** neurogenesis, cerebral cortex, immature neurons, human brain, doublecortin

## Abstract

This work provides evidence of the presence of immature neurons in the human brain, specifically in the layer II of the cerebral cortex. Using surgical samples from epileptic patients and post-mortem tissue, we have found cells with different levels of dendritic complexity (type I and type II cells) expressing DCX and PSA-NCAM and lacking expression of the mature neuronal marker NeuN. These immature cells belonged to the excitatory lineage, as demonstrated both by the expression of CUX1, CTIP2, and TBR1 transcription factors and by the lack of the inhibitory marker GAD67. The type II cells had some puncta expressing inhibitory and excitatory synaptic markers apposed to their perisomatic and peridendritic regions and ultrastructural analysis suggest the presence of synaptic contacts. These cells did not present glial cell markers, although astroglial and microglial processes were found in close apposition to their somata and dendrites, particularly on type I cells. Our findings confirm the presence of immature neurons in several regions of the cerebral cortex of humans of different ages and define their lineage. The presence of some mature features in some of these cells suggests the possibility of a progressively integration as excitatory neurons, as described in the olfactory cortex of rodents.

## Introduction

During the last decades different laboratories, including our own, have characterized a peculiar population of neurons confined to the layer II of the adult rodent piriform cortex (PCX) (Seki and Arai, 1991; Nacher et al., 2002; Gómez-Climent et al., 2008; Xiong et al., 2008; Bonfanti and Nacher, 2012; Rubio et al., 2015; Rotheneichner et al., 2018; Benedetti et al., 2020). Most, if not all, of these neurons are postmitotic cells of embryonic origin that remain in a prolonged immature state until adulthood (Gómez-Climent et al., 2008; Rubio et al., 2015; Yang et al., 2015). On the basis of their size and morphology, these immature neurons have been classified as: (a) tangled (type I) cells, which display a small soma and short neurites with irregular trajectories, usually restricted to layer II, and (b) complex (type II) cells with a larger soma, which usually display one or two long dendrites extending into layer I; neurons with intermediate characteristics have also been found, suggesting a progressive transition between these two cell types (Gómez-Climent et al., 2008; Luzzati et al., 2009; Gomez-Climent et al., 2010).

Neurodevelopmental markers such as doublecortin (DCX) and the polysialylated form of the neural adhesion molecule (PSA-NCAM) are commonly used to identify this population of cells (see Bonfanti and Nacher, 2012 for review). In most of these immature neurons, the simultaneous expression of DCX and PSA-NCAM and the lack of expression of mature neuronal markers can be considered reliable evidence of an immature neuronal phenotype. The vast majority express molecules exclusively found in pallial-derived excitatory neurons (Gómez-Climent et al., 2008; Luzzati et al., 2009; Rubio et al., 2015) and lack typical interneuronal markers (Gómez-Climent et al., 2008; Luzzati et al., 2009; Rubio et al., 2015). Recent works have demonstrated that the immature neurons of the PCX integrate progressively into the cortical circuitry as excitatory neurons during adult life (Rotheneichner et al., 2018; Benedetti et al., 2020). However, their precise function remains an open question.

Immature neurons, similar to those in the rodent PCX, have also been described in adult cortical regions of species with larger and gyrencephalic brains: rabbits (Luzzati et al., 2009), cats (Cai et al., 2009; Varea et al., 2011), and primates (Cai et al., 2009; Zhang et al., 2009). PSA-NCAM and/or DCX expressing cells, have been observed in the entorhinal cortex of infants (Dhúill et al., 1999) and in the upper border of the neocortical layer II of individuals of different ages (Cai et al., 2009; Sorrells et al., 2021). Additionally, despite the overall absence of glial markers in the immature neurons of rodents (Gómez-Climent et al., 2008; Xiong et al., 2008; Rubio et al., 2015), some astrocytes expressing DCX have been described in the human neocortex (Verwer et al., 2007; Bloch et al., 2011). However, the distribution, fine structure, or neurochemical phenotype of the immature neurons in the adult human brain have not been still studied in detail. Therefore, the aim of the present work was to study these parameters using surgical samples from epileptic patients and *post-mortem* tissue. Additionally, to evaluate the putative impact of epilepsy on this population of immature neurons, we used a rat lithium-pilocarpine model to analyze changes in the distribution and morphology of the immature neurons in the PCX layer II.

## Materials and Methods

### Human Samples and Histological Procedures

#### Human Tissue Collection

*Post-mortem* and post-operative neurosurgical specimens were collected for this study. Frozen, 14 μm-thick, coronal sections containing the ventral region of the temporal lobe were obtained from the Stanley Foundation Neuropathology Consortium. This collection consists of 60 individuals divided into four groups: (a) normal control subjects (*n* = 15) and patients with (b) bipolar disorder (*n* = 15), (c) major depression without psychotic features (*n* = 15) and (d) schizophrenia (*n* = 15) (Group demographic summaries can be found in Table 1). Seventeen post-operative neurosurgical samples from the frontal, temporal, parietal, and occipital lobes, obtained with informed consent, were collected from patients with epilepsy from the Hospital La Fe (for clinical and demographic data see Table 2).

**TABLE 1:** Clinical and demographical data of the Stanley Neuropathology Consortium.

	MD	BD	SCHZ	Controls
N	15	15	15	15
Age years, mean (SEM), (range)	46.5 (2.4), (30–65)	42.3 (3.0), (25–61)	42.3 (3.0), (25–61)	48.1 (2.8), (29–68)
Sex male/Female	8/7	9/6	9/6	9/6
PMI, hours, mean (SEM), (range)	27.5 (2.8), (7–47)	32.5 (4.2), (13–62)	33.7 (3.8), (12–61)	23.7 (2.6), (8–42)
Tissue pH, mean (SEM), (range)	6.2 (0.06), (5.8–6.5)	6.2 (0.06), (5.8–6.5)	6.2 (0.07), (5.8–6.5)	6.3 (0.06), (5.8–6.5)
Suicide	7	9	4	0
Alcohol use	5	7	5	2
Psychosis	0	11	15	0
Brain weight, gr, mean (SEM), (range)	1,462 (37), (1,240–1,740)	1,441 (44), (1,130–1,690)	1,471 (28), (1,270–1,640)	1,501 (42), (1,305–1,840)
Duration, years, mean (SEM), (range)	12.7 (2.9), (1–42)	20.1 (2.5), (6–43)	21.3 (3.0), (5–45)	

*BD, bipolar disorder; MD, major depression; PMI, post mortem interval; SCHZ, schizophrenia.*

**TABLE 2:** Clinical and demographical data of neurosurgical samples collected.

N	Age	Gender	Clinical history	Brain region
1	5	M	Epilepsy intraoperative resection	TL
2	5	F	Epilepsy intraoperative resection	FL
3	43	F	Epilepsy intraoperative resection	FL
4	48	M	Epilepsy intraoperative resection	FL
5	34	F	Epilepsy intraoperative resection	FL
6	25	F	Epilepsy intraoperative resection	FL
7	55	M	Epilepsy intraoperative resection	TL
8	62	F	Epilepsy intraoperative resection	TL
9	44	M	Epilepsy intraoperative resection	TL
10	42	F	Epilepsy intraoperative resection	TL
11	47	M	Epilepsy intraoperative resection	TL
12	28	M	Epilepsy intraoperative resection	TL
13	45	M	Epilepsy intraoperative resection	TL
14	37	M	Epilepsy intraoperative resection	TL
15	20	M	Epilepsy intraoperative resection	FL
16	46	M	Epilepsy intraoperative resection	PL
17	13	F	Epilepsy intraoperative resection	OL

*FL, frontal lobe; OL, occipital lobe; PL, parietal lobe; TL, temporal lobe.*

### Human Samples and Histological Procedures

#### Stanley Foundation Neuropathology Consortium

All brains underwent clinical neuropathological examination by two neuropathologists and none of them demonstrated evidence of neurodegenerative changes or other pathological lesions. Sections were unfrozen and fixed by immersion in 2.5% PFA solution in lysine-phosphate buffer pH 7.4 for 20 min at room temperature. After fixation, sections were washed in PB pH 7.4 and processed for immunohistochemistry. All the studied sections passed through the procedures simultaneously to minimize any difference arising from histochemical and immunohistochemical protocols. From the Stanley Neuropathology Consortium, we only considered 20 individuals, in which positive cells could be detected in the layer II; the rest of samples were excluded due to low histological quality in this layer. The samples of the Consortium included exclusively the temporal cortical lobe (containing the entorhinal and perirhinal cortices and the fusiform gyrus).

#### Post-operative Neurosurgical Samples

After surgical resections, samples were immediately immersed in 10% formalin and post-fixed at 4°C in fresh PFA 4% in 0.1 M PB, pH 7.4. Twenty-four hours later, tissue was washed in 0.1 M PB under gentle shaking for 24 h at 4°C. Coronal sections were sliced with a Leica VT1200s microtome at a thickness of 50 μm and kept until use in PB 0.1 M with sodium azide 0.05% as preservative.

### Immunohistochemistry for Conventional Light Microscopy

For conventional PSA-NCAM immunohistochemistry, *post-mortem* tissue samples were processed on slides, while post-operative tissue samples were processed “free-floating.”

All tissue sections (*post-mortem* and post-operative) were incubated 15 min in an antigen unmasking solution (0.01 M citrate buffer, pH 6) at 100°C. After cooling down sections to room temperature, they were incubated with 3% H_2_O_2_ in PBS for 10 min to block endogenous peroxidase activity. After this, sections were treated for 1 h with 5% NDS (Jackson Laboratories) in PBS with 0.2% Triton-X100 (Sigma-Aldrich) and were incubated overnight at room temperature in anti-PSA-NCAM antibody (1:1,400; Abcys, Paris, France) (Supplementary Table 1). After washing, sections were incubated for 1 h with donkey anti-mouse IgM biotinylated antibody (1:250; Jackson ImmunoResearch Laboratories) (Supplementary Table 1), followed by an avidin-biotin-peroxidase complex (ABC; Vector Laboratories, Peterborough, United Kingdom) for 30 min in PBS. Color development was achieved by incubating with 3,3′-diaminobenzidine tetrahydrochloride (Sigma-Aldrich) and 0.033% hydrogen peroxide in PB for 4 min. PBS containing 0.2% Triton-X-100 and 3% NDS was used for primary and secondary antibodies dilution.

### Immunohistochemistry for Confocal Microscopy

Fluorescence immunohistochemistry was performed as described above but omitting the endogenous peroxidase block. Samples were incubated for 48 h at 4°C with a mixture of primary antibodies (Supplementary Table 1). After washing, sections were incubated for 2 h (room temperature) with a specific secondary antibody cocktail (Supplementary Table 1). Thereafter, sections were washed in PB 0.1 M and mounted on slides in the case of the “free-floating” sections. To remove autofluorescence caused by aldehyde fixation samples were treated with Sudan Black B (SBB; Sigma-Aldrich) (Schnell et al., 1999; Oliveira et al., 2010). To this end, slides were incubated with a solution of 0.1% SBB in 70% ethanol for 20 min followed by three washes for 5 min in PBS with 0.02% Tween 20 (Sigma-Aldrich). Finally, all sections were washed in PB 0.1 M and coverslipped using DakoCytomamation fluorescent mounting medium (Dako).

#### Antibody Characterization

All antibodies used in this study were previously validated by the manufacturers and extensively used by other researchers on rodent and human brain tissue (manufacturers’ information). Additionally, we tested each antibody in 4% PFA fixed rodent brain slices before conducting the experiments. They gave a regional and cellular immunolabeling distribution comparable to that of their respective antigens with the same or other equally selective antisera. Additionally, in order to confirm that some of the immunostaining was not produced by the secondary antibodies or by the immunocytochemical protocol, we omitted primary antibodies or substituted them by NDS. These controls resulted in a complete absence of immunostaining in every case. Below there is a detailed description of the antibody characterization. In the case of the anti-DCX and anti-PSA-NCAM antibodies we were able to run additional controls for their specificity.

The anti-PSA-NCAM antibody was generated against rat embryonic spinal cord membranes. The specificity of immunostaining in our tissues was tested in 3 different ways, as described before (Gómez-Climent et al., 2008): (a) three different anti-PSA antibodies were used, and they rendered exactly the same pattern of immunostaining; (b) pretreatment of the antibody with a-2,8-linked sialic polymer (colominic acid, Sigma) overnight, or the primary antibody omission during the IHC prevented all labeling in the rat piriform cortex layer II and in the human cortex; (c) PSA immunohistochemistry of sections from animals injected intracerebrally with EndoN resulted in a complete absence of PSA immunostaining in the area affected by the injection.

The DCX antibodies detected endogenous levels of the protein DCX. They were generated against a synthetic peptide corresponding to human DCX. Antibodies are purified by protein A and peptide affinity chromatography. The staining pattern seen in this study using the DCX-rabbit polyclonal antibody (4604S; Cell Signaling) is consistent with other studies in human tissue (Liu et al., 2008, 2018; Sorrells et al., 2019). Antibody specific signal was proven using either DCX knockout mice (Kappeler et al., 2006) or double labeling with a DCX-mouse monoclonal antibody (SC27139; Santa Cruz). In the case of the DCX knockout mice all labeling was absent from layer II. When using both primary antibodies we observed complete overlap of expression in layer II cells. The polyclonal antibody showed the most extensive labeling of cells and processes, for this reason it was used across all samples for the descriptive and quantitative analysis.

The CTIP2 protein is a reported synonym for the human gene BCL11B, encoding BAF chromatin remodeling complex subunit BCL11B. The full-length protein is 95,519 Da, with 2 identified isoforms. When used in WB, this monoclonal antibody recognized 2 bands representing CTIP2 at about 120 kDa (manufacturer’s datasheet). The CTIP2 antibody has been validated for its use in human with more than 50 citations (manufacturer’s information).

The CUX1 antibody was raised against recombinant fragment corresponding to amino acids 521–621 of Human CUTL1. This monoclonal antibody reacts with human tissue (manufacturer’s information) and recognizes a single band of predicted molecular weight of 164 kDa on WB (manufacturer’s datasheet).

The Ank-G monoclonal antibody is raised against a synthetic peptide derived from the spectrin-binding domain of Ank-G human origin. The antibody recognized 2 bands, at 480 and 270 kDa, which are thought to correspond to the 2 forms of ankyrin on WB of whole-cell lysates from a human cell line (manufacturer’s data sheet). It is recommended for detection of Ank-G of mouse, rat and human origin by WB, immunohistochemistry and immunoprecipitation (manufacturer’s information). The staining pattern in the current study was consistent with other studies in humans (Tian et al., 2014; Szegedi et al., 2020).

The GAD67 antibody clone 1G10.2, reacts with the 67 kDa isoform of glutamate decarboxylase of rat, mouse and human origins. According to the manufacturers, no detectable cross reactivity with GAD65 has been found by WB on rat brain lysate when compared to blots probed with the antibody AB1511 (Merck-Millipore) that reacts with both GAD65 and GAD67 isoforms. It has been validated for its use in WB, immunohistochemistry and immunoprecipitation for the detection of GAD67 and has more than 75 product citations, including some in humans (manufacturer’s information). The staining pattern in the current study is consistent with other studies in humans (Fish et al., 2008; Neman et al., 2014).

The GFAP antibody was generated against a recombinant full-length protein corresponding to human GFAP. It recognizes a band of approximately 55.48 kDa on WB (manufacturer’s datasheet). The GFAP antibody was validated in human tissue with more than 50 citations (manufacturer’s information).

The IBA1 antibody has been generated against a synthetic peptide from mouse. It recognizes a band of approximately 10–15 kDa (predicted molecular weight: 17 KDa) (manufacturer’s datasheet). The use of this antibody in human brain tissue was previously described by Mendes et al. (2018).

The NeuN monoclonal antibody is routinely evaluated by manufacturers by means of IHC on brain tissue (avian, chicken, mouse, rat and human) and its use have been validated by published studies for immunocytochemistry, immunohistochemistry and immunoprecipitation, including some in human tissue and WB (manufacturer’s information). The staining pattern in the current study is consistent with other studies in human tissue (Talbot et al., 2004; Martí-Mengual et al., 2013).

The TBR1antibody has been generated against a synthetic peptide corresponding to aa 50–150 conjugated to keyhole limpet hemocyanin from mouse. When used in WB, it recognizes a single band of approximately 74 KDa (predicted molecular weight: 74 KDa). This antibody has been validated by IHC for its use in human tissue (manufacturer’s information).

The VGLUT1 antibody is generated against a synthetic peptide from rat VGLUT1 protein with no overlap to VGLUT2. Western blot reveals a single band at approximately 60 kDa (Melone et al., 2005). Moreover, pre-absorption of VGLUT1 antibody with immunogen peptide eliminates all immunostaining (manufacturer’s product information). VGLUT1-immunopositive puncta were also abolished in sections from VGLUT1-KO animals (Siembab et al., 2016). This antibody has been used in previous studies in human brain tissue (Talbot et al., 2004; Alonso-Nanclares and Defelipe, 2005).

The GluN1 antibody is generated against a synthetic peptide from rat GluN1 protein. Pre-absorption of GluN1 antibody with immunogen peptide (Alomone labs) eliminates all immunostaining (manufacturer’s and own results in our tissue).

#### Observation and Quantification of Labeled Cells

A Nissl stain with toluidine blue in alternate series of sections was used to determine the boundaries and layers of the cerebral cortex regions. To evaluate the presence of PSA-NCAM expressing cells in layer II, 5 sections per subject were randomly selected and observed with an Olympus CX41 microscope under bright-field illumination at 40× magnification.

For the characterization of the neurochemical phenotype of cells in layer II, sections were observed under a confocal microscope (Leica TCS-SPE). Z-series of optical sections (1 μm apart) covering the whole thickness of the histological sections were obtained using sequential scanning mode. Photographs were taken at 20×, 40×, or 60× magnification and in some cases were digitally zoomed at 0.5× to 2×. All images were processed with FIJI/ImageJ software (Schindelin et al., 2012). Twenty-five cells, in a minimum of 5 sections per subject, were randomly selected and manually counted to determine the percentage of colocalization between the different markers. The density of DCX + and NeuN + cells was estimated manually from 10 single confocal planes (275 × 275 μm) randomly chosen from layer II, taken from 3 different patients.

#### Electron Microscopy of Doublecortin Immunoreactive Cells

For transmission electron microscopy, we used sections from 2 biopsy cases from the ones used for immunofluorescence (4% PFA postfixation). Two sections from each individual were selected and rinsed twice in PB (2 × 30 min) to remove sodium azide. Then the sections were cryoprotected for 30 min by using 25% sucrose, 10% glycerol in 0.01 M PB. Then they were freeze-thawed three times on an aluminum boat on liquid nitrogen to enhance antibody penetration. The sections were incubated in 1% sodium borohydride in PB for 30 min to reduce aldehyde groups. After washing, the sections were incubated in 0.3% hydrogen peroxide in PB for 15 min. Then the sections were incubated in Tris buffer 0.5 N pH 7.2 for 30 min. As a blocking solution for immunohistochemistry, we used 0.1% fish gelatin, 1% normal donkey serum in PB for 1 h. We incubated with anti-DCX rabbit primary antibody we used Rabbit anti human-DCX antibody (Supplementary Table 1) in PB containing 0.1% fish gelatin, 1% NDS, 0.01 sodium azide in PB for 5 days at 4C, followed by biotinylated donkey anti-rabbit secondary antibody (Supplementary Table 1) for 2 h. Finally, sections were incubated in 1:200 ABC (Vector Laboratories, Peterborough, United Kingdom) for 1.5 h. Color development was achieved using 0.05% DAB (Sigma-Aldrich) and 0.01 hydrogen peroxide for 20 min. After each step sections were washed three times in PB (3 × 30 min).

After extensive washing in PB, sections were postfixed in 2% glutaraldehyde in PB for 15 min, this step does not increase the quality of the ultrastructure but toughens the section for the inclusion processing. Sections were incubated in 1% osmium tetroxide in PB, then they were dehydrated in increasing concentrations of cold ethanolic solutions up to 70% (30, 50, and 70%; 10 min each). Then, sections were incubated in 1% uranyl acetate in 70 ethyl alcohol for 1 h at 4C. The dehydration of the sections continued using 70%, 90%, 2 X absolute ethanol (10 min each) and then cleared twice in propylene oxide (10 min each). Subsequently, the sections were incubated overnight in Durcupan epoxy resin (Fluka). Next day the sections were flat-embedded between slide and coverslide and polymerized at 60°C in an oven for 24 h.

Sections were observed under a light microscope. Selected DCX cells were photographed and then, after coverslide removal, they were excised using a surgical scalpel and re-embedded for ultramicrotomy. Series of consecutive ultrathin sections (60 nm) were obtained using an ultramicrotome (EM UC6, Leica) on formvar coated nickel grids. Grids were contrasted using 1% uranyl acetate in 70% ethanol for 1 min and then in a 0.2% lead citrate contrasting solution for 5 min (Venable and Coggeshall, 1965). The sections were observed using a transmission electron microscope (Jeol JEM 1010) at 60 Kv. The cells were identified by correlation with the light microscopy and by the DAB electron density. Images were taken using a digital camera (AMT 8Mpx). Putative synaptic contacts were considered when they appeared in at least two consecutive sections, using as criteria the presence of associated densities and synaptic cleft.

### Lithium-Pilocarpine Epilepsy Model in Rats

For the Lithium-pilocarpine model, 8 adult male Sprague-Dawley rats (3 months old, 250–300 g; Charles River Laboratories, MA, United States) were used. The rats were housed per groups in a standard environment (12 h light/dark cycle) with ad *libitium* access to food and water. All animal experimentation was conducted in accordance with the Directive 2010/63/EU of the European Parliament and of the Council of 22 September 2010 on the protection of animals used for scientific purposes and was approved by the Committee on Bioethics of the University of Valencia. Every effort was made to minimize the number of animals used and their suffering.

Rats received pilocarpine (30 mg/Kg, s.c; Sigma-Aldrich) (treated, *n* = 4) or saline (control, *n* = 4) 6 h later the administration of the lithium chloride (3 mEq/Kg, s.c; Sigma-Aldrich). The onset of fulminant status epilepticus occurred 15–30 min after pilocarpine administration. Animals were then administered diazepam (10 mg/kg s.c.; Henry Schein, NY, United States) approximately 90 min after seizures onset. After 72 h, all animals were perfused transcardially under deep chloral hydrate anesthesia, first with saline and then with 4% PFA in PBS 0.1 M, pH 7.4. Thirty minutes after perfusion, the brains were extracted from the skull and cut in coronal 50 μm-tick sections using a vibratome (Leica VT 1000E). Slices were collected in 10 subseries and stored in 0.1 M PB with sodium azide 0.05% as preservative at 4°C. They were used for conventional PSA-NCAM immunohistochemistry. Sections were processed “free-floating” using the ABC method as described for the human samples.

### Quantification and Statistical Analysis

All slides were coded prior to analysis and the codes were not broken until the experiment was finished. Statistical analyses were performed using GraphPad Prism 9 software (GraphPad Software Inc.). Mean ± SEM was determined and the resulting values were analyzed by unpaired Student’s *t*-tests after checking the normality and homoscedasticity of the data.

## Results

### Morphology and Distribution of Doublecortin Immunoreactive Cells in the Human Cerebral Cortex Layer II

**FIGURE 1:**
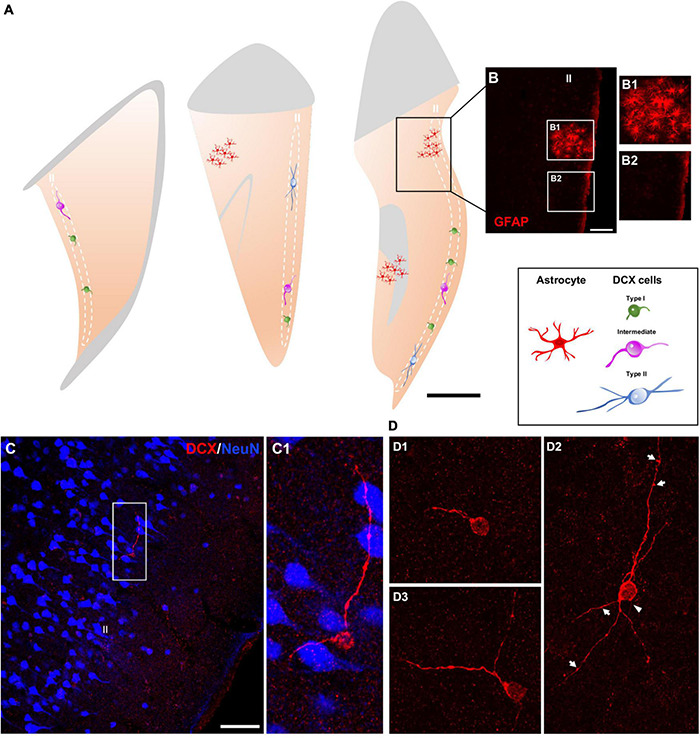
Distribution and morphology of DCX expressing cells in the human cerebral cortex layer II. **(A)** Schemes of 3 representative 50 μm coronal sections of temporal cortex, showing areas with reactive astrogliosis (red) and DCX immunoreactive cells in the layer II displaying different morphologies (type I, green; intermediate, magenta; type II, blue). **(B)** Confocal image of the squared area in **(A)**, showing GFAP immunofluorescence. **(B1,B2)** Are higher magnifications of the squared areas in **(B)** showing regions with **(B1)** or without **(B2)** reactive astrocytes. **(C)** Double-immunofluorescence for DCX (red) and NeuN (blue) in the temporal cortex. Note a DCX + cell with its soma located in the layer II and lacking a NeuN immunoreactive nucleus. NeuN immunohistochemistry was used to define the six-layered cerebral cortex. **(C1)** Is a higher magnification of the squared area in **(C)**. **(D)** 2D projections of confocal stacks (5 confocal planes separated by 1 μm) showing DCX expressing cells: type I **(D1)**, type II **(D2)**, and intermediate **(D3)**. A thin process resembling an axon (arrowhead) and sparse protrusions similar to stubby dendritic spines (arrows) can be observed in the type II cell shown in figure **(D2)**. In **(A)** white and gray matters are indicated with gray and light-orange colors, respectively. Scale bars: 30 mm for **(A)**; 70 μm for **(B)**; 35 μM for **(B1,2)**; 70 μm for **(C)**; 20 μm for **(C1)**; 30 μm for **(D1–3)**. All schemes and confocal images in this figure were from neurosurgical samples.

Immunohistochemical analyses revealed a population of DCX + cells in the layer II of the cerebral cortex of humans (Figure 1). No qualitative differences were found in the distribution or morphology of these cells between the different groups of the postmortem samples from the Stanley Neuropathology Consortium (see section “Materials and methods”). In the neurosurgical samples, an anti-glial fibrillary acidic protein (GFAP) antibody was used to discard areas with reactive astrogliosis (Figures 1A,B); all analyses were performed outside these areas.

DCX + cells were found dispersed within the layer II (identified with NeuN immunolabeling), preferentially in the upper region limiting with layer I, of all cerebral lobes: frontal, temporal, parietal, and occipital (Figure 1C). These cells appeared frequently isolated, although occasionally could be detected in pairs. The quality of the samples from the Stanley Neuropathology Consortium only allowed for the detection of scarce isolated cells due to the poor histological preservation of the tissue in the outer layers of the cerebral cortex. Consequently, these samples were used only for double labeling experiments directed to analyze the phenotype of these cells and not for quantitative analyses. The histological preservation in the neurosurgical samples was much better and allowed an estimation of the linear density of these cells, particularly in the temporal lobe from most of the samples were taken (3.0 ± 1.4 per mm). We have also performed a densitometric analysis in the temporal lobe comparing the presence of DCX + cells with that of NeuN + ones in the layer II. The density of DCX + cells was 0.006 ± 0.002 cells/mm^2^, while those of NeuN + was considerably higher (0.8 ± 0.2/mm^2^).

The DCX expressing cells in the human cerebral cortex layer II were similar morphologically to those recently described in the neocortex of several gyrencephalic mammalian species (La Rosa et al., 2019). They can be classified as type I: small (soma diameter between 3 and 9 μm) cells showing a single short process restricted to layer II (Figure 1D1) and type II: large (soma diameter between 9 and 17 μm) multipolar cells with longer dendritic arborization, frequently oriented in parallel to layer II (Figure 1D2). These type II cells displayed a thin process resembling an axon and had both apical and basal dendrites. These dendritic arbors were not very complex and frequently displayed varicosities and protrusions, a few of which resembled stubby spines (length ≤ 1 μm) (Figure 1D2). Both DCX + cell types were present in the layer II (type I: 65.3 ± 10.32% and type II: 12.94 ± 5.0%), together with cells displaying intermediate morphologies (21.6 ± 6.27%), which usually had a soma diameter between 9 and 11 μm, a thin basal process and only 1 or 2 short dendrites without spines or excrescences (Figure 1D3).

### The Doublecortin Expressing Cells in the Human Cerebral Cortex Layer II Are Immature Neurons

**FIGURE 2:**
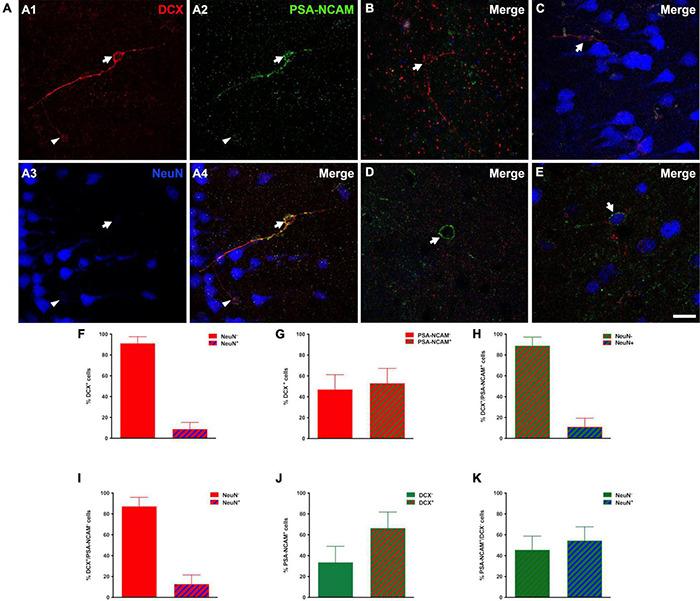
Immature phenotype of DCX immunoreactive cells in the human cerebral cortex layer II. **(A–E)** Triple-immunofluorescence for DCX (red), PSA-NCAM (green) and NeuN (blue) in the layer II of the occipital lobe. **(A1–4)** Cells colocalizing DCX and PSA-NCAM and lacking NeuN immunoreactivity in their nuclei. Note the presence of a large (type II) DCX + /PSA-NCAM + cell (arrow) and a smaller one (type I, arrowhead). **(B)** DCX positive type II cell lacking PSA-NCAM and NeuN expression. **(C)** Type II cell expressing DCX and NeuN and lacking PSA-NCAM immunoreactivity. **(D)** PSA-NCAM positive cell lacking DCX and NeuN expression. **(E)** Cell immunoreactive for PSA-NCAM and NeuN and lacking DCX expression. **(F,G)** Graphs showing the percentage of DCX + cells displaying NeuN immunoreactive nuclei **(F)** or co-expressing PSA-NCAM **(G)**. **(H)** Graph showing the percentage of DCX + /PSA-NCAM + cells displaying a NeuN + nucleus. **(I)** Graph showing the percentage of PSA-NCAM + /DCX- cells displaying a NeuN immunoreactive nucleus **(J)** Graph showing the percentage of PSA-NCAM + co-expressing DCX. **(K)** Graph showing the percentage of PSA-NCAM + /DCX- cells displaying a NeuN immunoreactive nucleus. Error bars represent the mean ± SEM. The images are 2D projections of 5 consecutive confocal stacks (1 μm apart). Scale bar: **(A–C)** 30 μm, **(D,E)** 20 μm. All confocal images in this figure were from neurosurgical samples.

Most (91.17 ± 6.37%) DCX immunoreactive cells lacked expression of NeuN in their nuclei and thus could not be considered mature neurons (Mullen et al., 1992). Only a small population (8.83 ± 6.37%) displayed NeuN immunoreactivity, mainly very well developed/large type II cells (Figures 2A–C,F).

Several studies, in both gyrencephalic mammals and rodents, have demonstrated that the co-expression of DCX and PSA-NCAM may be considered a solid evidence of an immature neuronal phenotype (Bonfanti and Nacher, 2012; Varea et al., 2012; König et al., 2016). In *post-mortem* and surgical samples, our results with conventional light microscopy revealed a similar distribution of PSA-NCAM and DCX expressing cells in the cerebral cortex layer II. More than half of DCX expressing cells (53.0 ± 14.2%) coexpressed PSA-NCAM (Figures 2A,G) and only a very low percentage of these double labeled cells coexpressed NeuN (11.1 ± 8.2%) (Figures 2H,I). Moreover, many PSA-NCAM + cells (66.4 ± 15.3%) expressed DCX (Figures 2D,J). However, more than half of PSA-NCAM + /DCX- cells (54.43 ± 13.1%) were NeuN + and most likely mature interneurons (Figures 2E,K). In order to know whether the distribution or the density of immature neurons could be altered in our samples from epileptic patients, we studied an animal model of this neurological disorder. The rats submitted to the experimental epilepsy protocol still displayed considerable numbers of PSA-NCAM expressing cells in the PCX layer II, although in a reduced density, when compared to control animals (Supplementary Figure 1).

None of the DCX + cells, including type II cells bearing a thin process resembling an axon, expressed Ank-G. This protein is a marker of the axonal initial segment (AIS), which is a structure related to neuronal maturation (Gutzmann et al., 2014; Bolós et al., 2019) and not present in immature neurons of cortical layer II in rodents (Rotheneichner et al., 2018). The pyramidal neurons in layers II or III expressed Ank-G in their AIS (data not shown).

**FIGURE 3:**
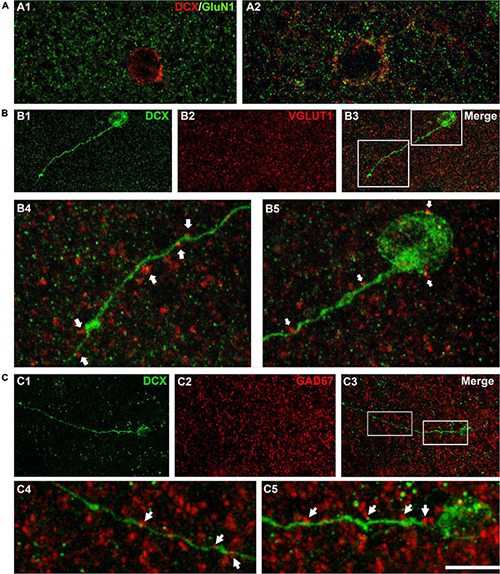
Expression of NMDA receptors (GluN1) and apposed puncta expressing VGLUT1 and GAD67 on DCX immunoreactive cells. **(A)** Confocal microphotographs of the temporal cortex showing the expression of GluN1 in small type I **(A1)** and larger type I DCX + cells **(A2)**. **(B)** DCX immunoreactive type II cell (green) in the occipital cortex layer II showing puncta expressing the excitatory marker VGLUT1 (red) apposed to its soma and dendrite (arrows). **(C)** DCX immunoreactive type II cell (green) in the temporal cortex layer II. Note the presence of GAD67 expressing puncta (red) apposed to its soma and dendrite (arrows). **(A1)** Is a single confocal plane, **(A2,B,C)** are 2D projections of 4 **(A2)** 9 **(B)** and 12 **(C)** consecutive confocal stacks (0.38 μm apart). Scale bar: 10 μm for **(A,B4,B5,C4,C5)**; 40 μm for **(B1–3,C1–3)**. All confocal images in this figure were from neurosurgical samples.

We have also studied the expression of the NMDA receptor subunit GluN1 (Figure 3A) since this subunit is absent from immature neurons both in the dentate gyrus (Nácher et al., 2007) and in the PCX (Gómez-Climent et al., 2008) of adult rats. Our observations confirm that this subunit is not expressed in the smallest type I cells (Figure 3A1) but can be found in larger type I cells and type II cells (Figure 3A2).

As another indicator of neuronal maturation, we studied the presence of puncta expressing the inhibitory and excitatory synaptic markers GAD-67 and VGLUT1 located in close apposition to the dendrites and the somata of DCX immunopositive cells. These puncta were absent from the small type I cells, but were detectable, although at low densities, on the large type I cells and were more abundant on type II cells (Figures 3B,C). This suggests the presence of inhibitory and excitatory synapses on type II immature neurons.

Under electron microscopy type II cells had small fusiform somata with scarce organelles. The nucleus had abundant peripheral heterochromatin. The plasma membrane was in direct contact with axons and dendrites; no covering by astroglial lamellae was observed (Supplementary Figure 2). The dendrites were difficult to follow far from the soma since they were thin and, although the DAB gave them electron density, the amount of electron dense elements in the ischemic neuropil impaired the task. Therefore, the search for putative synaptic contacts was limited to the soma of the cells.

On the soma we have identified some scarce contacts, which may correspond to synapses. However, the poor ultrastructural preservation generated by the PFA immersion fixation of the tissue did not allow definitive identification. These contacts were identified mostly by the presence of a putative synaptic cleft and the presence of small associated densities (about 150 nm; Supplementary Figures 2B,C arrows), suggesting the presence of synapses. The synaptic vesicles were not easily visible due to the poor ultrastructural preservation generated by the PFA immersion fixation of the tissue. On the other hand, dense core vesicles were better preserved and helped identification when present (Supplementary Figure 2B, barbed arrow). Some *puncta adherentia* were found connecting the cells to neuropil elements, but they were clearly differentiated from synapses since they presented thick dense plaques on both sides (not shown).

### The Immature Neurons of the Human Cerebral Cortex Layer II Belong to the Excitatory Neuronal Lineage

**FIGURE 4:**
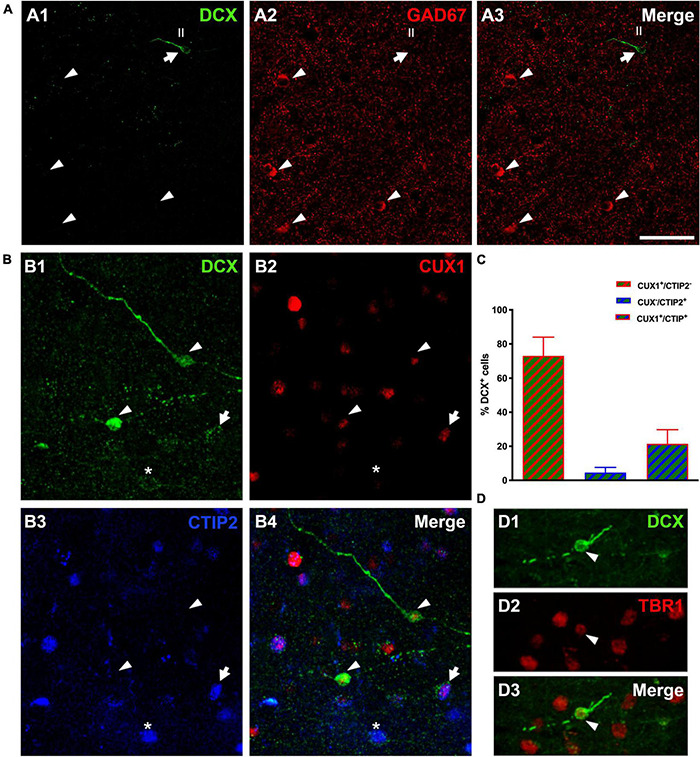
Lineage of immature neurons in the human cerebral cortex layer II. **(A)** Panoramic view of 2D projections (10 consecutive confocal stacks, 1 μm apart) showing the lack of co-localization between DCX (green) and GAD-67 positive cells (red). Note a single DCX large type I immunopositive cell (arrow) in the temporal cortex layer II, lacking GAD67 expression in its soma. GAD-67 immunoreactive cells (arrowheads) can be detected in the deeper layers of the cortex. **(B)** DCX + type II cells (green) co-expressing CUX1 (red, arrowheads), CTIP2 (blue, asterisk) or both transcription factors (arrow). **(C)** Graph showing the percentage of DCX cells expressing each transcription factor. Error bars represent the mean with ± SEM. **(D)** Immunofluorescence staining for DCX (green) and transcription factor TBR1 (red); arrowhead points to a type II cell co-expressing both markers. Images are 2D projections of 10 **(A,B)** and 15 **(D)** consecutive confocal stacks (1 μm apart). Scale bar: 50 μm for **(A)**, 25 μm for **(B)**; 10 μm for **(D)**. All confocal images in this figure were from neurosurgical samples.

The analysis of DCX expressing neurons in the cerebral cortex layer II revealed that none of them co-expressed GAD67, a marker of interneurons (Figure 4A). In the mammalian CNS, the transcription factors CUX1 and CTIP2 are almost exclusively expressed by pyramidal neurons of the upper cortical layers (Cubelos et al., 2010). Therefore, a triple immunohistochemistry for these 2 transcription factors and DCX was performed to confirm the excitatory lineage of the immature neurons. Our data revealed that many DCX expressing cells expressed CUX1 (73.0 ± 11.0%), a lower percentage of DCX cells expressed **only** CTIP2 (4.57. ± 3.0%) and a similar percentage expressed both transcription factors (21.43 ± 8.32%) (Figures 4B,C). Furthermore, we analyzed the expression of the neuron specific transcription factor T-box brain 1 (TBR1), exclusively expressed by pallial derived excitatory neurons (Hevner et al., 2001), and found that all DCX expressing cells analyzed expressed TBR1 (Figure 4D).

### The Immature Neurons of the Human Cerebral Cortex Layer II Are Not Glial Cells

**FIGURE 5:**
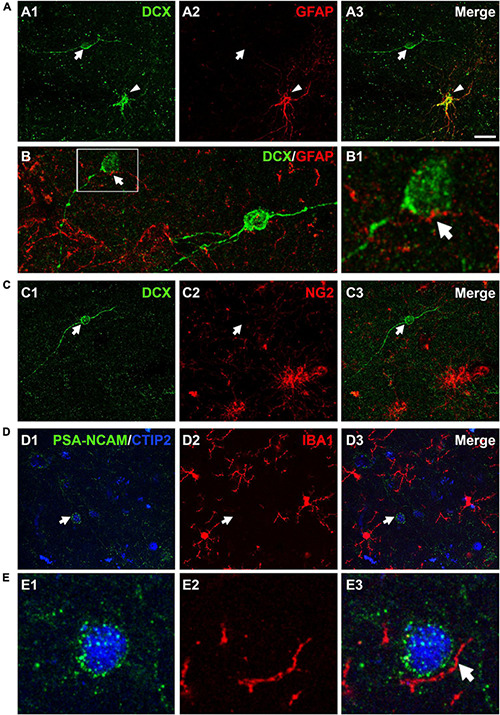
Expression of glial cell markers in the immature neurons of the human cerebral cortex layer II. **(A)** Double DCX (green) and GFAP (red) immunohistochemistry. Arrow points to a type II DCX immunopositive cell devoid of GFAP expression. Arrowhead indicates a DCX/GFAP double labeled cell. **(B)** Confocal reconstruction of a type II DCX immunoreactive cell (green) contacted by different astroglial processes (red). **(B1)** Depicts a higher magnification view of the squared area in **(B)**, in which the arrow indicates a GFAP immunopositive process closely apposed to the DCX + soma. **(C)** DCX immunopositive type II cell (green, arrow) devoid of NG2 expression (red, arrow). Note the presence of 2 NG2 immunoreactive cells lacking DCX expression in the lower portion of the figure. **(D)** No co-localization was observed between a PSA-NCAM + /CTIP-2 + double labeled neuron (arrow) and IBA1 immunopositive cells (red). The transcription factor CTIP2 was used to confirm the excitatory phenotype of PSA-NCAM immunoreactive neurons in layer II. **(E)** High magnification view showing an IBA1 + process (arrow) closely apposed to the soma of a PSA-NCAM expressing cell. All images are 2D projections of 15 confocal stacks (1μm apart). Scale bar: 30 μm for **(A,C,D)**; 10 μm for **(B)**; 5 μm for **(B1)** and 6 μm for **(E)**. All confocal images in this figure were from neurosurgical samples.

Different reports have described the presence of DCX immunopositive cells coexpressing GFAP in the cerebral cortex of human and non-human primates (Verwer et al., 2007; Bloch et al., 2011). Our results showed that the vast majority (97.2 ± 1.0%) of DCX expressing cells in layer II did not express GFAP; only a very low percentage (2.7 ± 1.0%) of these cells co-expressed this astroglial-specific intermediate filament (Figure 5A). Of note, these DCX + /GFAP + cells had the typical morphology of hypertrophic/reactive astrocytes. PSA-NCAM + cells never had the morphology of astrocytes. GFAP immunohistochemistry also showed astroglial processes apposed to the somata and the dendrites of DCX expressing cells in layer II (Figure 5B). Interestingly, these processes were abundant on type I cells, whereas they were scarce on type II cells, similar to what has been described in the PCX of rodents (Gómez-Climent et al., 2008).

Since a small proportion of immature neurons in the adult rodent PCX layer II was found positive for NG2, a marker of polydendrocytes or oligodendrocyte progenitor cells (Gómez-Climent et al., 2008; Rubio et al., 2015), we also performed a DCX/NG2 double fluorescence immunohistochemistry. Our results excluded this eventuality, since none of the DCX expressing cells in layer II were positive for NG2 (Figure 5C).

In order to know whether some of the immature neurons in the human cerebral cortex layer II corresponded to microglial cells and to investigate whether there was a spatial relationship between them, we used an anti-IBA1 antibody. Due to species incompatibility between the anti-IBA1 and the anti-DCX antibody, we visualized the immature cells using a combination of anti-PSA-NCAM and anti-CTIP2 antibodies, avoiding thus the detection of PSA-NCAM + mature interneurons. None of the PSA-NCAM^+^/CTIP2^+^ cells expressed IBA1 (Figure 5D). Interestingly, we observed processes expressing IBA1 closely apposed to the somata of PSA-NCAM^+^/CTIP2^+^ cells (Figure 5E).

## Discussion

In this work, we have provided evidence of a population of immature neurons in the human cerebral cortex layer II, have described their distribution in all the cortical lobes and have defined their phenotype and lineage.

The present study generalizes the spatially restricted description of these cells provided by previous studies in humans to the whole cortex: frontal, temporal, parietal, and occipital lobes. A single band of PSA-NCAM expressing cells was described for the first time in the entorhinal cortex in *post-mortem* samples of early infants (Dhúill et al., 1999). DCX expressing cells have been also found in adult human cortical samples of a wide age range (Cai et al., 2009); these cells, which showed a morphology resembling those described in our study, were identified only in the temporal and frontal cortices. DCX + cells were also found in these cortices in pediatric epilepsy surgical samples (Srikandarajah et al., 2009) and DCX + and PSA-NCAM + cells have been recently described in the parahippocampal gyrus of humans of different ages (Sorrells et al., 2021). By analogy with what occurs in other large-brained mammals (La Rosa et al., 2020), in which the number of immature neurons significantly increases in association with brain size, it may be presumed that the total number of immature neurons in the human brain must be high if one considers the linear density of these cells and their density per area when compared with NeuN + neurons (around 0.7%).

We did not observe notable differences in the distribution of immature cells between different age-groups, although the number of our samples was very limited. By contrast, in a previous study a differential DCX expression pattern was described in different ages (Srikandarajah et al., 2009): DCX + cells were identified mainly in biopsies from pediatric patients with epilepsy and focal cortical dysplasia, but they were not found in significant numbers in other epilepsy pathologies or in the cortex of *post-mortem* tissue of non-epileptic patients of similar age. Furthermore, DCX + cells were virtually absent from adult surgical samples and from *post-mortem adult* control tissue. One possible explanation for these discrepant results could be the use of paraffin embedding in their study, which could have adversely affected the immunogenicity of the samples. It has to be noted that in our neurosurgical samples, the analysis of the distribution of immature neurons, was conducted outside the areas affected by the epileptic seizures, which were specifically isolated by the use of an anti-GFAP antibody. Notwithstanding, the analysis of the rat lithium-pilocarpine epileptic model also indicates that epilepsy does not appear to affect critically this population of immature neurons. Similarly, a recent report studying surgical samples of human epileptic hippocampi reported substantial numbers of PSA-NCAM immunoreactive neurons in the subgranular zone (Seki et al., 2019).

In general terms, the neurosurgical samples showed better histological quality than the samples collected from the Stanley Neuropathology Consortium. The use of surgical biopsies provides many advantages, especially because it avoids sample fixation in formalin for prolonged periods, which negatively influences the detection of molecules by IHC procedures (Flor-García et al., 2020). Moreover, *post-mortem* tissue is highly vulnerable to several factors that interfere with the molecular preservation, the processing of these samples and their histological examination (Boekhoorn et al., 2006; Ferrer et al., 2008).

The immature phenotype of cells described in the human cerebral cortex layer II was defined by the expression of DCX and PSA-NCAM, which are commonly used to identify immature neurons in the adult CNS (Gómez-Climent et al., 2008; Cai et al., 2009; Luzzati et al., 2009; Varea et al., 2011; Rubio et al., 2015; La Rosa et al., 2020). Given the poor functioning of numerous antibodies on human tissue, the detection of DCX and PSA-NCAM had to be validated through different immunohistochemical controls. Due to the lack of blocking peptides, we have had to use alternate methods to demonstrate antibody specificity, particularly in the case of anti-DCX antibodies. We used 2 commercially available antibodies, which were previously applied in other human studies showing DCX expression in the neocortex, hippocampus and amygdala (Liu et al., 2018; Sorrells et al., 2019; Flor-García et al., 2020). Additionally, we have tested them in DCX-KO mice. The anti PSA-NCAM antibody has been also previously used in human studies and its specificity tested using different methods (Varea et al., 2007, 2012).

The morphology of the DCX + cells described in this study (type I and type II) resembled that previously described in gyrencephalic mammals (La Rosa et al., 2020) and are similar to those described as tangled and complex cells in the rodent PCX (Rotheneichner et al., 2018). Moreover, DCX + cells with intermediate morphological characteristics were also described, suggesting that these cells do not belong to 2 separated populations, but represent a single type that undergoes a progressive process of maturation, similar to what we have found recently in rodents (Rotheneichner et al., 2018). However, it has to be noted that the dendritic arbor of the type II cells in humans is, in general, less complex than that of the rodent cells. This could indicate that these cells cease to express immature neuronal markers earlier than in rodents or that they do not reach a similar level of differentiation.

Interestingly, for the first time in humans, we have observed the presence of protrusions that resemble stubby dendritic spines and thin axon-like basal processes on DCX + type II cells. It has to be noted that these spine-like protrusions are scarce. However, their density and morphology is similar to that present in complex (type II cells) DCX + neurons in rodents (Gómez-Climent et al., 2008). Experiments with DCX-GFP inducible transgenic mice have demonstrated that, after losing DCX expression these cells develop into typical spiny excitatory neurons (Rotheneichner et al., 2018) and this may also be the case in humans. The presence of these structures may indicate the appearance of nascent synaptic contacts. In fact, we have detected puncta expressing excitatory and inhibitory synaptic markers in close apposition to the dendrites and somata of type II cells. However, this is no definitive proof of the presence of synapses. Particularly, since these immature neurons have an excitatory phenotype, perisomatic VGLUT1 + puncta most likely represent synapses with the neuropil and not with the DCX + somata (Alonso-Nanclares and Defelipe, 2005). In rodents, ultrastructural analyses revealed asymmetric synapses on these protrusions resembling spines (Gómez-Climent et al., 2008), but unfortunately, we have not been able to study these structures in humans. Consequently, we have no definitive evidence of their nature. We also failed to find expression of the cytoskeletal scaffold protein Ank-G, which is normally expressed by mature AIS and is essential for its assembly and long-term maintenance (Hedstrom et al., 2008). Therefore, it is likely that these cells are not yet fully differentiated. The presence on type II immature cells of an extension resembling an axon was previously observed in rats (Gómez-Climent et al., 2008) and cats (Cai et al., 2009). We have examined selected DCX + type II cells under the electron microscope: The ultrastructural features of the DCX + neurons in the human cortex also resemble those found in immature DCX + neurons present in other parts of the brain, including the layer II of the piriform cortex of rodents (Gómez-Climent et al., 2008). These cells present scarce cytoplasm and organelles (including mitochondria) and the chromatin present abundant peripheral heterochromatin. In addition, these cells are not isolated by glial shafts. This is similar to what we described previously in rats: tangled (type I cells) appear covered by glial processes but these apposed processes were less abundant in complex (type II) cells (Gómez-Climent et al., 2008). Unfortunately, the fixation of our samples did not allow the clear discrimination of synapses on these cells. We have only been able to study their somata, since, as described in results, their neurites were impossible to follow. We have identified perisomatic contacts suggestive of synapses. However, the ultrastructural preservation of the tissue did not allow their definitive discrimination or whether they establish asymmetric or symmetric contacts. The presence of dense core vesicles is very interesting and may suggest the presence of synaptic input from monoaminergic axons. In this line, a recent paper from our laboratory has shown that in the piriform cortex of adult rats, immature neurons express dopamine D2 receptors and dopaminergic axons are closely apposed to these cells (Coviello et al., 2020).

Most DCX expressing cells in layer II co-expressed PSA-NCAM and lacked NeuN immunostaining in their nuclei, confirming their immature phenotype. The expression of NeuN was only detectable in a small number of DCX + /PSA-NCAM + cells with larger size and lighter reactivity, whereas it was completely absent in cells with type I morphology. This may be considered as an indication of a maturational process from type I cells to the most complex type II cells, which could be one-step-behind virtually mature neurons. The lack of expression of GluN1 in most type I cells and its presence in type II cells can be also interpreted as an indication of neuronal maturation. This pattern of GluN1 expression during neuronal maturation has been already described in the PCX and the dentate gyrus of adult rats (Nácher et al., 2007; Gomez-Climent et al., 2011).

As in rodents (Gómez-Climent et al., 2008; Rubio et al., 2015), rabbits (Luzzati et al., 2009), and cats (Varea et al., 2011), the immature neurons in the human cerebral cortex did not co-express GAD67, a marker of GABAergic interneurons. In addition, the expression of specific transcription factors also confirmed their excitatory lineage. These immature cells expressed CUX1 and CTIP2, which are exclusively expressed by pyramidal neurons of the upper cortical layers (II-III-IV) (Cubelos et al., 2010; Li et al., 2010; Hulea and Nepveu, 2012; Lennon et al., 2017), and TBR1, which is specific for pallium-derived principal neurons (Hevner et al., 2001). This, together with the presence of puncta expressing inhibitory and excitatory synaptic markers apposed to the somata and dendrites of DCX + type II cells, suggest that these cells are slowly being integrated into the circuitry as excitatory neurons. This progressive integration of the immature neurons into the circuitry as principal neurons has been demonstrated recently in the PCX of adult mice (Rotheneichner et al., 2018; Benedetti et al., 2020).

Since we have not found type II cells with numerous spines, potential contacts or Ank-G expression; it is possible that the immature neuronal markers were down-regulated before that stage. However, it is also possible that these cells do not reach enough synaptic contacts or maturation to gain functional relevance and, contrary to what happens in rodents (Rotheneichner et al., 2018), they remain as static non-functional or poorly integrated cells.

The immature neurons of the human cerebral cortex layer II cannot be classified as astrocytes, polydendrocytes/oligodendrocyte precursor cells or microglial cells. Nevertheless, it is very interesting to note that astroglial and microglial processes were found in close apposition to the surface of the somata and dendritic processes. Similar results have been described in rodents (Gómez-Climent et al., 2008). A putative insulating role has been proposed for this glial coverage, which correlates well with the apparent lack of synaptic input in the most immature neurons (Bonfanti and Nacher, 2012). DCX/GFAP expressing cells could be found in many of our samples and had the morphology of hypertrophic astrocytes. However, they were not more abundant in our epilepsy material. Moreover, most of the reactive astrocytes found in the epileptic foci did not express DCX. Previous reports have also described the presence of DCX/GFAP immunoreactive cells in human and non-human primate cortex (Verwer et al., 2007; Bloch et al., 2011), although they appeared to be more abundant than in our study.

The function of this population of immature neurons in the adult brain remains still poorly understood. Since the distribution of these immature neurons in rodents was primarily described in the olfactory cortex (Gómez-Climent et al., 2008), the first plausible hypothesis was that these cells were involved in olfactive functions. Subsequently, considering the wider distribution of these cells in mammalian species with larger, gyrencephalic cortices, including humans, is tempting to speculate on their putative involvement in the regulation of higher-order brain integrative abilities (Bonfanti and Nacher, 2012; La Rosa et al., 2020). In connection to this, there are still many questions to be answered, especially regarding the involvement of these immature cells in intracortical circuits or the possibility that they may receive inputs from the thalamus or other extracortical sources. Since the connectivity of these cells remains still unclear, further studies combining immunohistochemical and tracing studies are needed to highlight these questions. These immature neurons may represent a *“reservoir”* of young plastic neurons that under physiological or pathological circumstances complete their differentiation program to be consequently recruited into the preexisting neural circuits (Bonfanti and Nacher, 2012; La Rosa et al., 2019, 2020).

## Data Availability Statement

The raw data supporting the conclusions of this article will be made available by the authors, without undue reservation.

## Ethics Statement

Ethical review and approval was not required for the study on human participants in accordance with the local legislation and institutional requirements. Written informed consent to participate in this study was provided by the participants’ legal guardian/next of kin. The animal study was reviewed and approved by Committee on Bioethics of the University of Valencia.

## Author Contributions

JN and SC designed the experiments. SC, YG, and PK performed the experiments and quantifications. AG provided the surgical samples. SC, CC, JB-I, EV, and JN wrote the manuscript. All authors revised, edited the final version, contributed to the article, and approved the submitted version.

## Conflict of Interest

The authors declare that the research was conducted in the absence of any commercial or financial relationships that could be construed as a potential conflict of interest.

## Publisher’s Note

All claims expressed in this article are solely those of the authors and do not necessarily represent those of their affiliated organizations, or those of the publisher, the editors and the reviewers. Any product that may be evaluated in this article, or claim that may be made by its manufacturer, is not guaranteed or endorsed by the publisher.
