# Supplementary material for: Phenotype and Distribution of Immature Neurons in the Human Cerebral Cortex Layer II
Source: Front Neuroanat. 2022 Apr 8;16:851432. doi: 10.3389/fnana.2022.851432 (PMC9027810; doi:10.3389/fnana.2022.851432)
Supplement: Supplementary file 1 [file Data_Sheet_1.pdf]

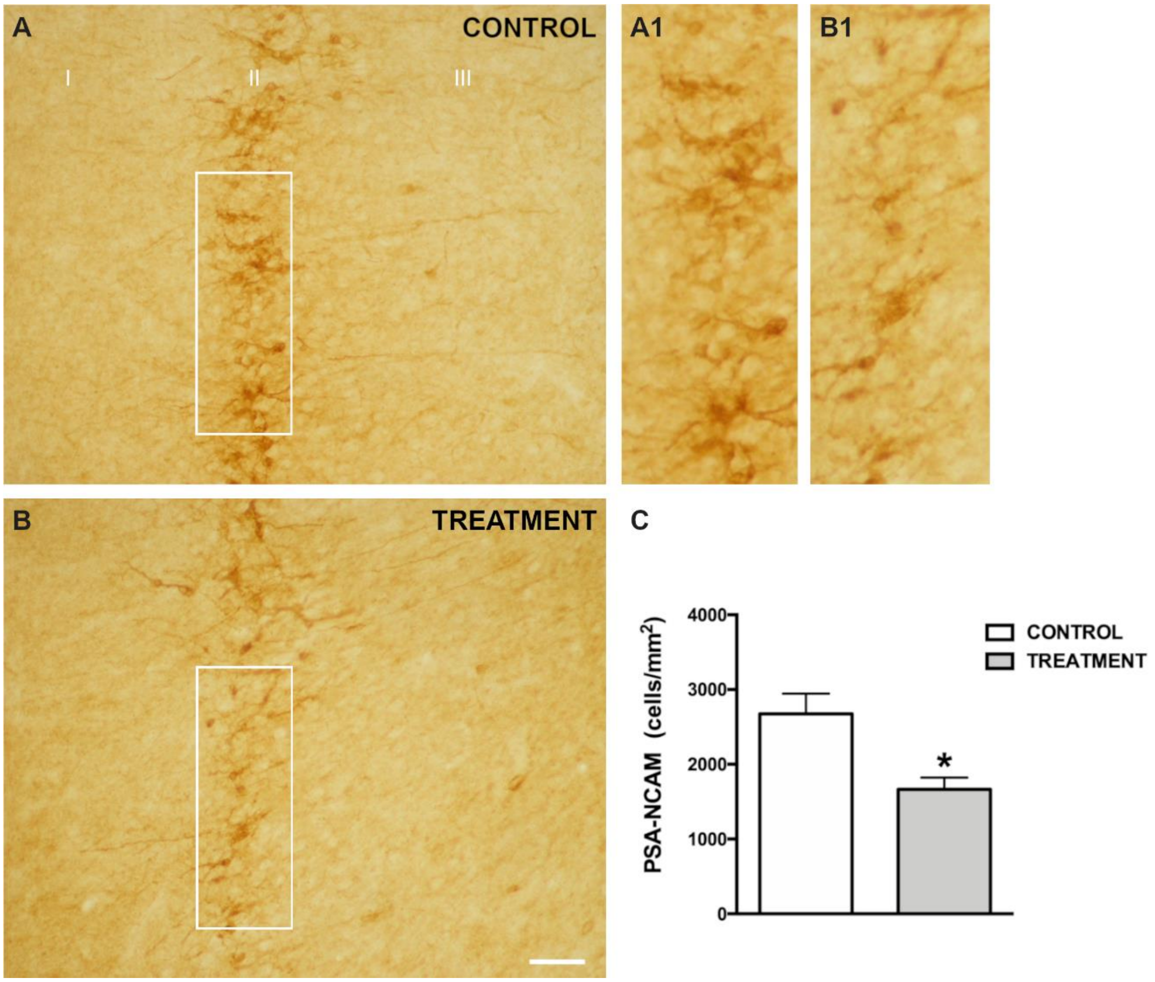

Fig. S1. Density of PSA-NCAM expressing cells in the piriform cortex layer II in the lithium-pilocarpine epilepsy model in rats. (A & B) Microphotographs showing PSA-NCAM immunoreactive cells in control (A) and after treatment with pilocarpine (B). (**A'-B'**) Higher magnification of the squared areas in A & B. **(C)** Graph representing changes in the density of PSA-NCAM expressing cells. Asterisks in treatment bars indicate statistically significant differences from control groups after unpaired Student's t-test; $p < 0.05$ (*). Scale bars: 200 µm for (A & B); 100 µm for (A1 & B1).

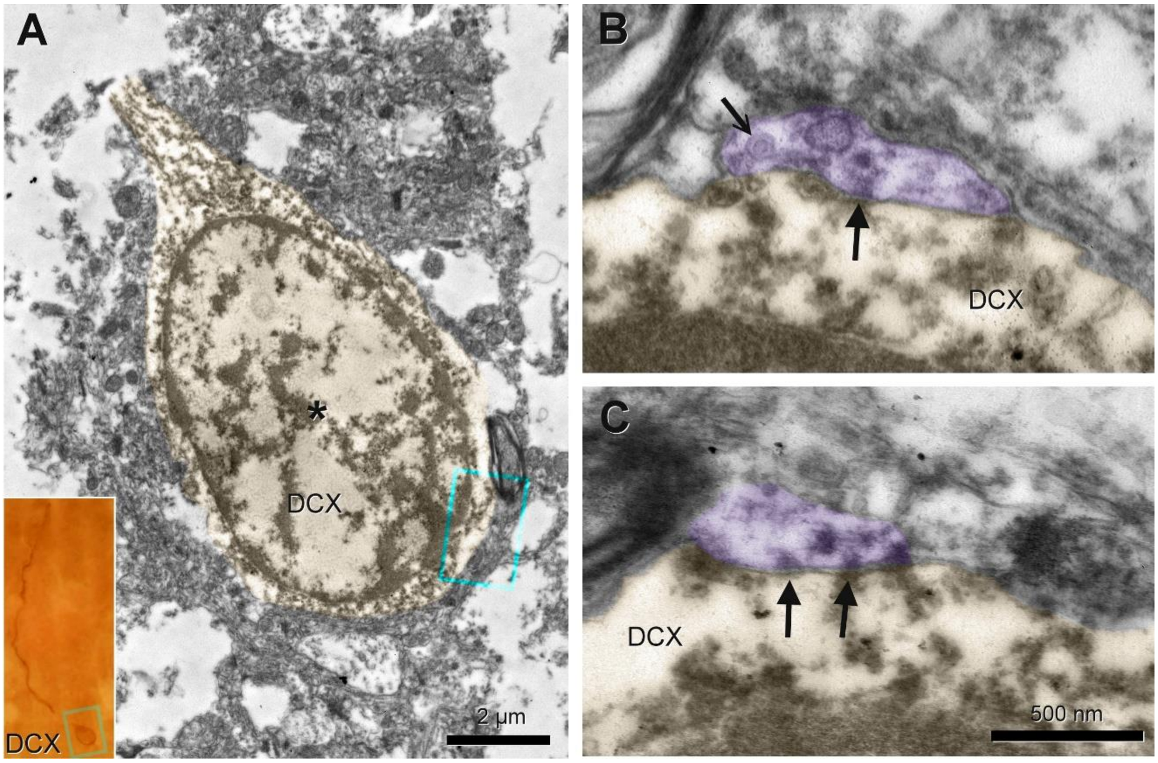

Fig. S2. Ultrastructure of type II DCX immunoreactive neurons in the human cerebral cortex layer II. (A) Field survey of a neuron (asterisk) identified on light microscopy (inset). The cell presents little cytoplasm and an ovoid nucleus with heterochromatin; organelles are scarce. (B & C) Two examples of asymmetrical synaptic contacts on the cell. Although the contacts are small (arrows), the postsynaptic density and the synaptic cleft are visible. On the presynaptic side, the clear vesicles cannot be accurately discriminated since membrane preservation is not optimal in PFA fixed tissue, but dense core vesicles are better preserved (barbed arrow). Scale bars: 2 μm for A, 500nm for B & C. False colors are used to identify the cell and the boutons. All images in this figure were from neurosurgical samples.

Table S1. **Primary and secondary antibodies used in the study**

Primary antibodies	***Anti***	***Host***	***Isotype***	***Dilution***	***Catalog number***	***Company***
	Ank-G	Mouse	IgG_1	1:200	SC12719	Santa Cruz
	CTIP2	Rat	IgG_{2A}	1:1000	Ab18465	Abcam
	CUX1	Mouse	IgG_1	1:100	Ab115854	Abcam
	DCX	Rabbit	IgG	1:500	4604S	Cell signaling
	DCX	Mouse	IgG_1	1:500	SC27139	Santa Cruz
	GAD67	Mouse	IgG_{2A}	1:1000	MAB5406	Sigma-Aldrich
	GFAP	Chicken	IgG	1:4000	Ab7260	Abcam
	IBA1	Rabbit	IgG	1:4000	Ab178846	Abcam
	NeuN	Mouse	IgG_1	1:1000	MAB377	Sigma-Aldrich
	PSA-NCAM	Mouse	IgM	1:1400	AbC0019	Abcys
	PSA-NCAM	Mouse	IgM	1:1400	MAB5324	Sigma-Aldrich
	TBR1	Rabbit	IgG	1:1000	Ab31940	Abcam
	VGLUT1	Guinea pig	IgG	1:1000	AB5905	Sigma-Aldrich
	GluN1	Rabbit	IgG	1:400	AGC-001	Alomone

Secondary antibodies	***Anti***	***Host***	***Conjugate***	***Dilution***	***Catalog number***	***Company***
	Chicken-IgY	Goat	AF555	1:400	A32932	ThermoFisher
	Chicken-IgY	Goat	CF488	1:400	20166	Biotium
	Guinea Pig-IgG	Donkey	CF555	1:400	20276	Biotium
	Mouse-IgG_1	Goat	CF633	1:400	20250	Biotium
	Mouse-IgG_1	Goat	AF555	1:400	A21127	ThermoFisher
	Mouse-IgG_{2A}	Goat	AF555	1:400	A21137	ThermoFisher
	Mouse-IgM	Goat	AF488	1:400	A21042	ThermoFisher
	Mouse-IgM	Goat	Biotin	1:400	A2338587	Jackson ImmunoResearch
	Rabbit-IgG	Goat	AF555	1:400	A32732	ThermoFisher
	Rabbit-IgG	Donkey	CF555	1:400	20038	Biotium
	Rat-IgG_{2A}	Goat	CF647	1:400	20843	Biotium
